# Antioxidant and Cryoprotective Effects of Bone Hydrolysates from Bighead Carp (*Aristichthys nobilis*) in Freeze-Thawed Fish Fillets

**DOI:** 10.3390/foods10061409

**Published:** 2021-06-18

**Authors:** Yiqi Zhang, Ye Dong, Zhiyuan Dai

**Affiliations:** 1 Key Laboratory of Aquatic Products Processing of Zhejiang Province, Institute of Seafood, Zhejiang Gongshang University, Hangzhou 310035, China; zhangyq@zjgsu.edu.cn (Y.Z.); dy12686@163.com (Y.D.); 2 Collaborative Innovation Center of Seafood Deep Processing, Dalian 116034, China

**Keywords:** bone hydrolysates, antioxidant activity, fillet, vacuum impregnation, freeze–thaw cycles

## Abstract

Bone hydrolysates from bighead carp (*Aristichthys nobilis*) were prepared using Protamex and Alcalase with degrees of hydrolysis (DH) of 5%, 10% and 15%. The antioxidant activity of bone hydrolysates was evaluated in vitro and then the hydrolysates with better antioxidant activity were used to immerse bighead carp fillets through a vacuum impregnation process at concentrations of 1% and 2%. Among the six hydrolysates, fish bone hydrolyzed with Protamex at DH 10% exhibited the highest ability to scavenge 1, 1-diphenyl-2-picrylhydrazyl (DPPH) (88.79%), 2, 2′-azino-bis-3-ethylbenzthiazoline-6-sulphonic acid (ABTS) (57.76%) and hydroxyl radicals (62.72%), as well as to chelate ferrous ions (91.46%). The hydrolysates effectively postponed freezing- and thawing-induced protein/lipid oxidation. Compared with the fillets without treatment, the impregnated fillets had higher sulfhydryl contents, greater Ca^2+^-ATPase activity, lower carbonyls and lower thiobarbituric acid-reactive substances (TBARS). Bone hydrolysates also have a positive effect on the texture and water-holding ability of freeze-thawed fish fillets. Fish bone hydrolysates of Protamex could serve as potential antioxidants to preserve fish fillets.

## 1. Introduction

Frozen storage is a common method for the long-term preservation of aquatic products. Microbial growth and endogenous enzyme activity can be effectively inhibited at low temperature, but protein/lipid oxidation still occurs [1], causing a deterioration in food quality, discoloration and protein denaturation, especially in conditions of temperature fluctuation or prolonged frozen storage [2,3]. To minimize the deteriorative reaction, some synthetic antioxidants (such as butylated hydroxytoluene (BHT) and butylated hydroxyanisole (BHA)) with toxicological and carcinogenic effects are being used [4,5]. Therefore, exploring natural additives for the prevention of protein/lipid oxidation represents a major challenge for the frozen aquatic products industry [6].

During the fish filleting process, about 70% of the fish becomes solid by-products with low economic value, such as fish skin, bones, scales, heads and viscera [7]. They contain high-quality protein and ought to be further exploited. About 30% of them, such as skin, bones or scales, can be used for the recovery of collagen/gelatin, which is widely used in food, biomedical and cosmetic industries [7]. Due to them being rich in glycine, proline and hydroxyproline, fish by-products could serve as raw materials for the production of bioactive peptides, which could be used as functional food ingredients [8]. The hydrolysates that are obtained, which usually contain lots of low molecular weight peptides, usually present more specific bioactivities than the native collagen/gelatin, such as antioxidant, antibacterial, dipeptidyl peptidase IV (DPP-IV) and angiotensin-converting enzyme (ACE) inhibitory activities [9,10]. 

Edible coatings based on natural antioxidant additives are a good choice for delaying the deterioration of fish fillets’ quality during storage [11,12]. In previous coating studies, only the surface parts of fish fillets were coated through dipping or spraying, thus greatly affecting the coating efficiency [13]. Vacuum impregnation (VI) is a promising technique with a high coating efficiency thanks to its combination of traditional impregnation processes and vacuum treatment [14]. It could directly accelerate the mass transfer between impregnation solutions and the food matrix due to deformation–relaxation phenomena and hydrodynamic mechanisms [15]. This process is applied to introduce functional compounds, such as antioxidants, cryoprotectants and bio-preservatives, into perishable foods to improve their quality [13,14].

Bighead carp (*Aristichthys nobilis*) is a commercially important freshwater fish in China; 3,101,637 tons were produced in 2019 [16]. During the production of bighead carp heads or fish fillets, large amounts of fish by-products are produced. Fish bones, as important parts of filleting coproducts, are good sources of high-value protein, bio-calcium and other nutrients [17,18]. Compared to the disposal or production of fishmeal or fertilizers [1], the preparation of functional peptides through controlled enzymatic hydrolysis would be a better choice to enhance the value of these by-products [19]. Recently, several protein hydrolysates with potential antioxidant activity in aquatic products were produced from fish by-products [10,20,21]. However, the effects of bone hydrolysates through VI on protein and lipid oxidation of frozen fillets remain unclear. Thus, the aim of the present work was to evaluate the in vitro antioxidant activity of protein hydrolysates from bighead carp bone with different degrees of hydrolysis (DH), as well as to investigate the potential inhibiting effects on protein and lipid oxidation of fish fillets during freeze–thaw cycles. The findings might help to provide a better by-product utilization strategy for the fish processing industry.

## 2. Materials and Methods

### 2.1. Materials and Reagents

Fresh bighead carp backbones were acquired at a local supermarket in Hangzhou, China. Alcalase 3.0T and Protamex were purchased from Novozymes (China) Biotechnology Co., Ltd. (Tianjin, China). Tea polyphenol was purchased from Fuzhiyuan Biotechnology Co., Ltd. (Jiangxi, China). Hippuryl-histidyl-leucine (HHL), bacitracin, aprotinin, cytochrome C, carbonic anhydrase, adenosine triphosphate (ATP), 5,5-dithio-bis (2-nitrobenzoic acid) (DTNB) and 2,4-dinitrophenylhydrazine (DNPH) were obtained from Sigma-Aldrich (Milwaukee, WI, USA). 2,2-Diphenyl-1-picrylhydrazyl (DPPH), 2,2-azino-bis(3-ethylbenzothiazoline-6-sulphonic acid) (ABTS) diammonium salt and 1,1,3,3-tetramethoxypropane (TEP) were obtained from Aladdin Reagents Co., Ltd. (Shanghai, China). Acetonitrile and trifluoroacetic acid (TFA) were of chromatographically grade. All other reagents with an analytical grade were purchased from Sinopharm Chemical Reagent Co., Ltd. (Shanghai, China).

### 2.2. Fish Bone Pretreatment

The carp backbone pretreatment was carried out on a steam explosion device (QBS-200B, Gentle Science and Technology Co. Ltd., Hebi, China) equipped with a 5 L chamber. The apparatus consisted of a steam generator, a receiver and a rapid-opening piston valve. About 300 g of fish bones were placed inside the vessel and treated with saturated steam at 0.6 MPa for 2 min, which was followed by an instantaneous release of pressure within 0.1 s. The exploded bones were collected and dried at 40 °C, crushed and stored at −20 °C.

### 2.3. Preparation of Fish Bone Hydrolysates

Bighead carp bone powders were placed into distilled water (6%, *w/v*) and hydrolyzed by Alcalase (pH 8.0) or Protamex (pH 7.0) at 50 °C with an enzyme/substrate (*w/w*) ratio of 1%. The hydrolysis reaction was performed in a double-walled glass reactor and the pH was maintained constant by addition of NaOH (0.1 M). Based on the pH-stat method, when the DH achieved 5%, 10% and 15%, respectively, the hydrolysates were immediately heated to 95 °C for 15 min to terminate the hydrolysis reaction, followed by centrifugation (Hettich Rotina 420R, Tuttlingen, Germany) at 8000 g for 15 min. The supernatant was frozen, lyophilized and stored at −20 °C for further study. Bone hydrolysate powders prepared using Alcalase or Protamex with a DH of 5%, 10% and 15% were referred to as HA5, HA10, HA15, HP5, HP10 and HP15, respectively.

### 2.4. Determination of Molecular Weight Distribution

The molecular weight (MW) distribution of the hydrolysates was analyzed on a TSK-Gel G2000 SW_XL_ column (7.8 mm × 300 mm, Tosoh, Tokyo, Japan) using a Waters e2695 HPLC system according to Zhang et al. [22]. The eluent was 45% acetonitrile solution containing 0.1% trifluoroacetic acid (TFA). The flow rate was 0.5 mL/min. The absorbance was monitored at 220 nm. The standards were shown as follows: carbonic anhydrase (29,000 Da), cytochrome C (12,400 Da), aprotinin (6500 Da), bacitracin (1422 Da) and HHL (429 Da). 

### 2.5. Determination of Antioxidant Activities of Hydrolysates

#### 2.5.1. DPPH Radical Scavenging Activity

The DPPH radical scavenging activity was measured according to Saisavoey et al. [20], with some modifications. Briefly, 1.0 mL hydrolysate (8.0 mg/mL) was added to 1.0 mL of 0.15 mmol/L DPPH solution in 95% ethanol. The mixture was shaken and kept for 30 min at room temperature and its absorbance was monitored at 517 nm. The DPPH radical scavenging activity of bone hydrolysates was expressed as follows:

DPPH radical scavenging activity (%) = [1 − (A_1_ − A_2_)/A_0_] × 100 (1)

where A_1_ represents the absorbance of a sample at 517 nm, A_2_ represents the absorbance of a sample blank without DPPH and A_0_ represents the absorbance of the control without a sample.

#### 2.5.2. ABTS Radical Scavenging Activity

The ABTS radical scavenging activity of the hydrolysates was measured according to Zheng et al. [23], with some modifications. ABTS radical stock solution was prepared by mixing 7 mmol/L ABTS with 140 mmol/L potassium persulfate and kept away from light at room temperature for 12–16 h. The ABTS solution was diluted with phosphate buffer (0.2 M, pH 7.4) to an absorbance of 0.70 ± 0.02 at 734 nm. A total of 150 μL of diluted ABTS solution was added to 50 μL of sample solution (1.2 mg/mL). After blending, the mixture was kept at room temperature for 6 min and its absorbance was then measured at 734 nm. The scavenging effect for ABTS radical was expressed as follows:

ABTS radical scavenging activity (%) = [1 − (A_1_ − A_2_)/A_0_] × 100 (2)

where A_1_ represents the absorbance of sample at 734 nm, A_2_ represents the absorbance of sample blank without ABTS and A_0_ represents the absorbance of the control without sample.

#### 2.5.3. Hydroxyl Radical Scavenging Activity

Hydroxyl radical scavenging activity was measured according to Lima et al. [24], with some modifications. The reaction mixture, containing 1.0 mL sample (8.0 mg/mL), 6.5 mL of distilled water, 0.5 mL of salicylic acid (10 mM) and 0.5 mL FeSO_4_ (10 mM), was mixed with 0.5 mL H_2_O_2_ (6 mM) and kept at 37 °C for 15 min. Its absorbance was determined at 510 nm. The hydroxyl radical scavenging activity was calculated as following equation:

Hydroxyl radical scavenging activity (%) = [1 − (A_1_ − A_2_)/A_0_] × 100 (3)

where A_1_ represents the absorbance of sample at 510 nm, A_2_ represents the absorbance of sample blank without H_2_O_2_ and A_0_ represents the absorbance of the control without sample.

#### 2.5.4. Ferrous Iron’s Chelating Activity

Ferrous iron’s chelating activity was determined according to Zhang et al. [25]. Briefly, 1 mL of 8 mg/mL sample solution was mixed with 3.7 mL distilled water and 0.1 mL FeCl_2_ (2 mM). Then, the mixture was reacted with 0.2 mL of ferrozine (5 mM) and incubated for 20 min at room temperature. Its absorbance was monitored at 562 nm. The control group was prepared by replacing the sample with distilled water. The metal chelating activity was calculated as follows:

Metal chelating activity (%) = (A_0_ − A_1_)/A_0_ × 100 (4)

where A_1_ represents the absorbance of a sample at 562 nm and A_0_ represents the absorbance of the control without a sample.

### 2.6. Fillets Preparation

A total of 20 bighead carp (2500 ± 200 g) were acquired at a local supermarket in Hangzhou, Zhejiang, China. After fish sampling, the carp were cleaned and cut into fillets (3 cm × 3 cm × 1.5 cm). The fillets (*n* = 80) were randomly separated into four groups. The four dipping solution were designed as follows: (i) the control group treated with distilled water (CK); (ii) the tea polyphenol group (0.1%, *w/v*) (TP); (iii) the bone hydrolysates group (1%, *w/v*) (HP1); and (iv) the bone hydrolysates group (2%, *w/v*) (HP2). The fillets were impregnated under 0.07 MPa with different solutions for 15 min, respectively. After treatment, the fillets were stored at −18 °C for 48 h and then thawed at 4 °C for 12 h. The samples were taken randomly for analysis after 1, 3, 5 and 7 freeze–thaw cycles. 

### 2.7. Preparation of Myofibrillar Protein

Myofibrillar protein was prepared according to Zhang et al. [26], with some modifications. Fish muscle (2 g) was homogenized (IKA T18 basic, IKA, Staufen, Germany) with 20 mL of chilled distilled water and centrifuged (Hettich ROTINA 420R, Tuttlingen, Staufen, Germany) at 8000 g for 10 min at 4 °C. The precipitate was dispersed in 20 mL 60 mM KCl-20 mM Tris-maleate (pH 7.0) and then centrifuged at 8000 g for 10 min. The obtained precipitate was further homogenized with 20 mL of 0.6 M KCl-20 mM Tris-maleate (pH 7.0), incubated at 4 °C for 1 h and then centrifuged for 10 min. The supernatant (8 mL) was then mixed with chilled distilled water (32 mL) and centrifuged for 10 min. The precipitate was finally dissolved with 0.6 M KCl (pH 7.0). The above experiments were all carried out under 4 °C. The myofibrillar protein concentration was determined using the Biuret method.

### 2.8. Determination of Sulfhydryl Groups

The sulfhydryl contents were determined according to Ellman [27], with some modifications. Myofibrillar protein solution (0.5 mL) was mixed with 4.5 mL of 0.2 M Tris-HCl (pH 8.0) containing 8 M urea, 3 mM EDTA and 1% sodium dodecyl sulfate (SDS). The mixture (4 mL) was added into 0.5 mL of 10 mM 5, 5-dithio-bis (2-nitrobenzoic acid) (DTNB) solution in 0.2 M Tris-HCl (pH 8.0), incubated at 40 °C for 25 min and then monitored by absorbance at 412 nm. A blank was prepared with 0.6 M KCl instead of the myofibrillar protein. The sulfhydryl group’s content was calculated using a molar extinction coefficient of 13,600 M^−1^ cm^−1^.

### 2.9. Determination of Protein Carbonyls

The protein carbonyls content was determined as described by Zhang et al. [28]. Briefly, 1 mL myofibrillar protein (2 mg/mL) was mixed with 1 mL of 10 mM 2, 4-dinitrophenylhydrazine (DNPH) (in 2 M HCl) in the dark for 1 h. Afterwards, the mixture was precipitated with 1 mL 20% trichloroacetic acid (TCA) and centrifuged (Fresco 21, Thermo Fisher Scientific, Pittsburgh, PA, USA) at 11,000 g for 10 min. The sediment was washed with 1 mL ethanol/ethyl acetate (1:1, *v*/*v*) three times to remove any free DNPH. The obtained precipitate was then dissolved in 3 mL guanidine hydrochloride (GuHCl) (6 M) and incubated at 37 °C for 15 min. The absorbance was measured at 370 nm. The protein carbonyl content was calculated using a molar extinction coefficient of 22,000 M^−1^ cm^−1^.

### 2.10. Determination of Ca^2+^-ATPase Activity

Ca^2+^-ATPase activity was determined according to Zhao et al. [13]. Briefly, the myofibrillar protein sample (0.4 mL) was mixed with the reaction solution (0.2 mL 0.1 M CaCl_2_, 0.2 mL 0.5 mM Tris maleate (pH 7.0), 3 mL distilled water and 0.2 mL 20 mM adenosine triphosphate (ATP)), kept for 5 min at room temperature and terminated by the addition of 2 mL 15% TCA. After centrifugation at 8000 g for 5 min, the inorganic phosphorus content in the supernatant was determined by an ammonium molybdate method. The Ca^2+^-ATPase activity was expressed as the inorganic phosphorus content generated per mg of myofibrillar protein per minute (μmol/mg/min).

### 2.11. Determination of Thiobarbituric Acid-Reactive Substances

Thiobarbituric acid-reactive substances (TBARS) were determined according to Kunyaboon et al. [29] and expressed as mg malonaldehyde (MDA) equivalents of kg^−1^ samples using a standard curve of 1,1,3,3-tetramethoxypropane (TEP). Briefly, 2 g of sample were homogenized with 10 mL of 10% TCA (*w*/*v*). After filtration, the supernatant (5 mL) was mixed with 5 mL of 20 mM thiobarbituric acid (TBA), heated at 95–99 °C for 20 min, then cooled to room temperature and monitored by absorbance at 532 nm. 

### 2.12. Determination of Drip Loss

Drip loss was determined according to Zhao et al. [13]. The weight of fish fillets was monitored before and after freeze–thaw cycles. The drip loss was calculated as follows:

Drip loss (%) = (W_1_ − W_2_)/W_1_ × 100 (5)

where W_1_ represents the weight of the fillets before freezing and W_2_ represents the weight of the fillets after each freeze–thaw cycle.

### 2.13. Determination of Textural Properties

The texture parameters of fish fillets, including hardness and springiness, were measured using a texture analyzer (TMS-Pro, Food Technology Co., Sterling, VA, USA) with a TMS 5 mm steel. The parameters were as follows: deformation rate of 60%, trigger force of 0.01 N and time interval of 5 s. A sample cube of 2 cm × 2 cm × 1.5 cm was used, and the process was repeated at least six times.

### 2.14. Statistical Analysis

Data were expressed as mean ± standard deviation (SD) of at least triplicates and analyzed by analysis of variance (ANOVA). Means were compared under the significance level of *p* < 0.05 using Duncan’s multiple range tests with SPSS 21.0 software (SPSS Inc., Chicago, IL, USA). 

## 3. Results and Discussion

### 3.1. Molecular Weight Distribution of Bone Hydrolysates

**Figure 1:**
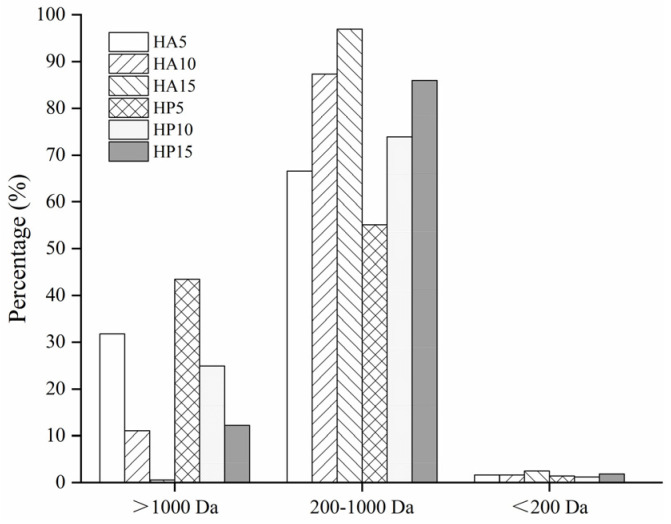
Molecular weight distribution of bone hydrolysates prepared by Alcalase and Protamex.

The antioxidant activity of protein hydrolysates is closely associated with molecular weight (MW) [30]. Accordingly, compared to those macromolecular peptides, the peptides containing fewer than 10 amino acid residues usually had higher antioxidant activities [1] due to their easier access to, and greater capacity to scavenge free radicals [31]. The MW distribution of fish bone protein hydrolysates of Alcalase (HA) and Protamex (HP) with a DH of 5%, 10% and 15% is shown in Figure 1. The samples were divided into three fractions (>1000 Da, 1000–200 Da, <200 Da). The proportion of high MW peptides in HP and HA decreased with increasing DH, which was accompanied by an increase in the proportion of low MW peptides. In addition, the amounts of low MW peptides in HA were higher than those in HP at the same degree of hydrolysis. This indicated that Alcalase had a higher hydrolysis efficiency on fish bone than Protamex. Protamex was a mixture of endo- and exoproteases, while Alcalase was comprised of endoproteases with broad specificities for peptide bonds [32]. Different proteases have different cleavage sites, resulting in differences in hydrolysis efficiency. As shown in Figure 1, all the proportions of MW (200–1000 Da) peptide in six samples accounted for more than 50% of the total. In particular, the HA sample with a DH of 15% contained the largest percentage (96.92%) of low MW substances between 200 and 1000 Da, followed by HA (10% DH) and HP (15% DH), with respective proportions of 87.31% and 85.95%. These hydrolysates had high percentages of low MW peptides, suggesting that they might have higher antioxidant properties.

### 3.2. In Vitro Antioxidant Activity of Bone Hydrolysates

The oxidative reaction is regarded as an important deterioration in quality in meat and meat products, and it is caused by non-microbial factors. Protein hydrolysates might delay lipid oxidation through several pathways, such as scavenging free radicals, inactivating reactive oxygen species (ROS), reducing hydroperoxides and chelating prooxidative transition metals [20]. To evaluate the antioxidant capacity of fish bone hydrolysates, the activities of DPPH, ABTS and hydroxyl radicals scavenging and metal chelation of the hydrolysates were determined in vitro. 

**Figure 2:**
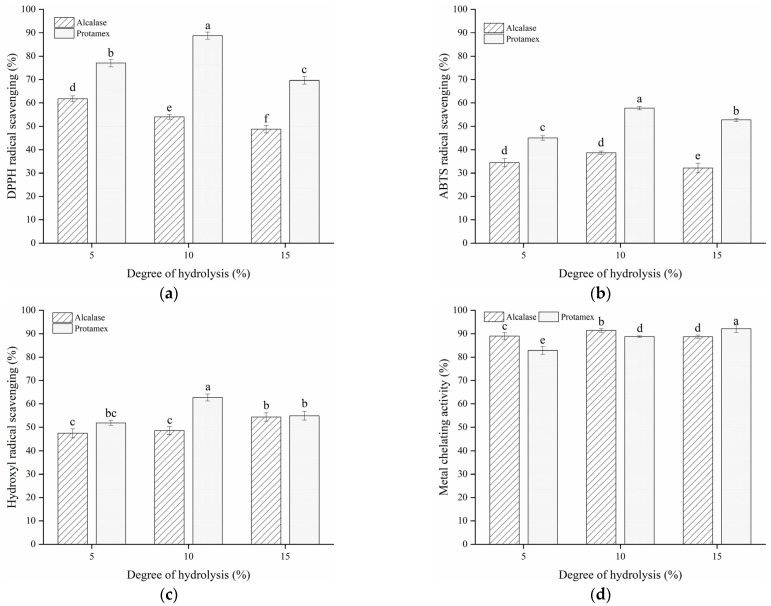
DPPH radical scavenging activity (**a**), ABTS radical scavenging activity (**b**), hydroxyl radical scavenging activity (**c**) and metal chelating activity (**d**) of bone hydrolysates prepared by Alcalase and Protamex. Different letters indicate the results differ significantly (*p* < 0.05).

The hydrolysates produced by different enzymes had different amino acid sequences, leading to varied DPPH radical scavenging abilities. As shown in Figure 2a, the hydrolysates obtained from Protamex with a DH of 5%, 10% and 15% showed a higher DPPH radical scavenging activity, with values ranging from 69.63% to 88.79% compared to those of Alcalase. DPPH in ethanol could be scavenged by substances with proton donors [30]. This indicates that HP (especially with a DH of 10%) might have some amount of special amino acids that function as proton donors, thereby exhibiting a higher DPPH radical quenching ability [33]. Additionally, the antioxidant activity of bone hydrolysates is highly related to the degree of hydrolysis. Although low molecular weight peptides exhibit stronger oxidant inhibition activity than larger ones, the antioxidant capability of peptides might decline when they are further hydrolyzed into free amino acids [31]. 

Bone hydrolysates of Alcalase and Protamex exhibited a similar tendency in arresting the ability of the ABTS radical (Figure 2b). The arresting ability of these hydrolysates both reached the maximum at a DH of 10% and decreased at a DH of 15%. HP with a DH of 10% displayed higher activity in relation to scavenging ABTS radicals, with a value of 57.76%, which is approximately 1.5 times higher than that of HA. This indicates that bone hydrolysates of Protamex with a DH of 10% are quite capable of retarding oxidation and could be used as a potential radical quencher in the preservation of food products.

Hydroxyl radical is recognized as damaging to biological molecules such as protein, DNA and lipids. As an important antioxidant, fish protein hydrolysate is used as an active free-radical scavenger to improve food quality [25]. The hydroxyl radical eliminating activity of HP was superior to HA with a DH of 5% and 10%, while no significant (*p* > 0.05) distinction was detected between the bone hydrolysates with a DH of 15% (Figure 2c). In addition, HP with a DH of 10% showed a higher hydroxyl radical scavenging activity (62.72%) than other hydrolysates, indicating that bone hydrolysates might be also potent hydroxyl radical removers in the defense against oxidation damage in meat. 

Similar to changes in radical scavenging activity, all hydrolysates of Alcalase and Protamex exhibited high Fe^2+^-chelating activity, with values over 80% (Figure 2d). The highest chelating activity belonged to HP with a DH of 15% (92.18%), followed by HA with a DH of 10% (91.46%). Compared to HA samples, the HP samples showed better antioxidant properties. Taken together, bone hydrolysates of Protamex with a DH of 10% were chosen as additives in freeze-thawed fish fillets.

### 3.3. Effect of Bone Hydrolysates on Protein Oxidation of Frozen Fish Fillets

#### 3.3.1. Sulfhydryl Content

**Figure 3:**
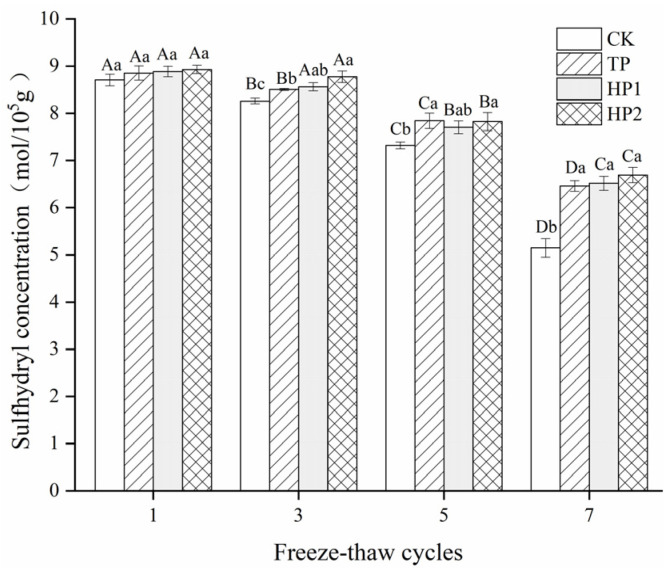
Changes in sulfhydryl contents of freeze-thawed fish fillets: CK: fillets in distilled water; TP: fillets in tea polyphenol; HP1: fillets in 1% bone hydrolysates; HP2: fillets in 2% bone hydrolysates. Different capital letters indicate the results that differ significantly in freeze–thaw cycles with the same treatment (*p* < 0.05). Different lowercase letters indicate the results that differ significantly with different treatments in the same freeze–thaw cycle (*p* < 0.05).

As a highly active group of proteins, the sulfhydryl group (SH) is easily oxidized in the presence of ROS, especially under temperature fluctuations [30]. The total sulfhydryl groups of bighead carp myofibrils generally declined with increasing freeze–thaw cycles (Figure 3). After one cycle, no obvious differences (*p* < 0.05) in sulfhydryl contents were observed among all the groups. However, from freeze–thaw cycles three to seven, sulfhydryl contents decreased the most in the CK group, while the values in the treated groups declined slowly, especially in the HP2-treated group. Compared with the first cycle, the losses in total sulfhydryl concentration were 40.91%, 27.00%, 26.66% and 24.97% in CK, TP, HP1 and HP2 groups, respectively, indicating that tea polyphenol and protein hydrolysates exhibited effective antioxidant activity against the oxidation of the SH group to disulfide bonds. The decrease in sulfhydryl groups during the frozen storage of seafood is mainly caused by the formation of disulfide bonds or inter- and intra-molecular cross-linking [3]. Gelatin hydrolysates from shark skin, with a DH of 10%, also showed protective effects on SH oxidation in surimi under different freeze–thaw cycles [34]. In this study, bone hydrolysates may exhibit a protective effect in the myofibrillar protein structure, hindering the overexposure of buried sulfhydryl and thus reducing the sulfhydryl oxidation reaction in muscles [1]. 

#### 3.3.2. Carbonyl Content

**Figure 4:**
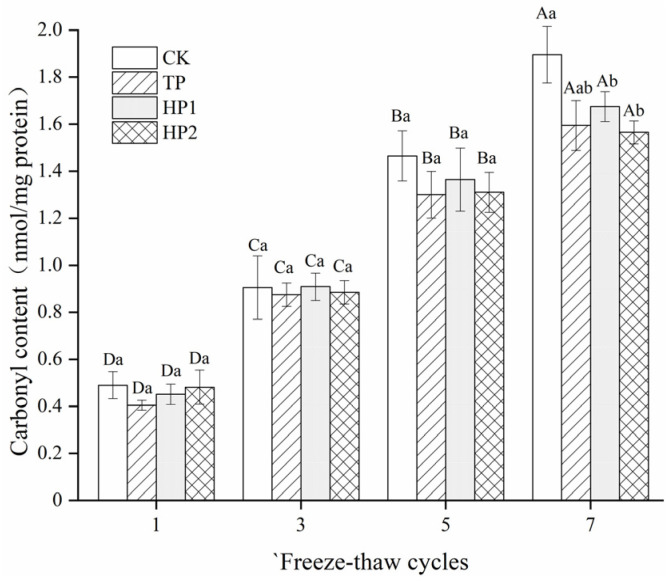
Changes in carbonyls’ contents in freeze-thawed fish fillets: CK: fillets in distilled water; TP: fillets in tea polyphenol; HP1: fillets in 1% bone hydrolysates; HP2: fillets in 2% bone hydrolysates. Different capital letters indicate the results that differ significantly in freeze–thaw cycles with the same treatment (*p* < 0.05). Different lowercase letters indicate the results that differ significantly with different treatments in the same freeze–thaw cycle (*p* < 0.05).

Generally, protein carbonyls are formed by the conversion of certain amino acid residues induced by protein oxidation [35]. There is a relationship between the formation of carbonyls and deteriorative processes, resulting in an off-flavor, as well as a loss of textural and nutritional qualities in meat during low-temperature storage. As shown in Figure 4, carbonyl contents all increased with the prolonged freeze–thaw cycles. Obviously, the carbonyl content in the CK group was the most elevated, as evidenced by a maximum carbonyl content with a 1.39 nmol/mg increase. The formation of carbonyls in fish fillets was significantly (*p* < 0.05) inhibited by bone hydrolysates and tea polyphenol, as evidenced by 1.2, 1.19 and 1.1 nmol/mg increases in TP, HP1 and HP2 groups, respectively. Furthermore, after the seventh cycle, the carbonyls’ value (1.56 nmol/mg) in the HP2 group was the lowest and was comparable to that (1.59 nmol/mg) in the TP group. Protein hydrolysates could form hydrogen bonds with amino acid residues to replace some water molecules around the protein surface, thus reducing protein oxidation [36]. In addition, the hydrolysates with good radical scavenging activity might also lower the carbonylation of certain amino acids through the binding of metal ions [37]. 

#### 3.3.3. Ca^2+^-ATPase Activity

**Figure 5:**
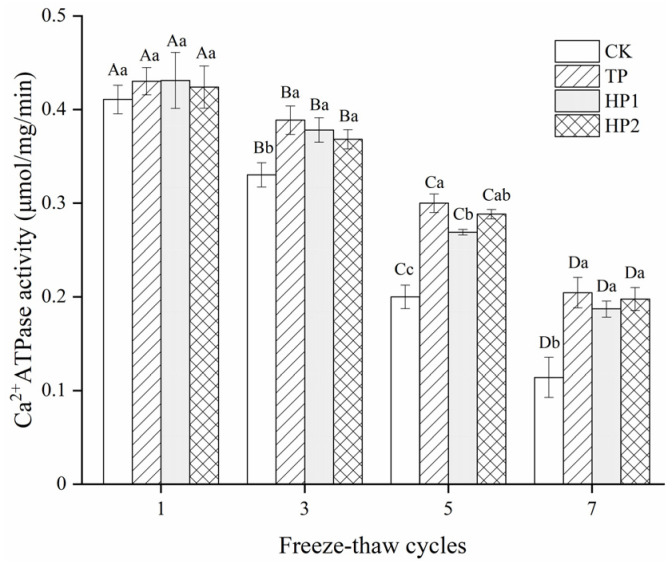
Changes in Ca^2+^-ATPase activity of freeze-thawed fish fillets: CK: fillets in distilled water; TP: fillets in tea polyphenol; HP1: fillets in 1% bone hydrolysates; HP2: fillets in 2% bone hydrolysates. Different capital letters indicate the results that differ significantly in freeze–thaw cycles with the same treatment (*p* < 0.05). Different lowercase letters indicate the results that differ significantly with different treatments in the same freeze–thaw cycle (*p* < 0.05).

Ca^2+^-ATPase activity serves as a good indicator of the integrity of myofibrillar proteins during frozen storage, which is associated with the reactivity of sulfhydryl groups in myofibrils. No significant (*p* > 0.05) change was observed in Ca^2+^-ATPase activity among samples after one cycle (Figure 5). However, the Ca^2+^-ATPase activity in fish fillets clearly decreased (*p* < 0.05) when subjected to multiple freeze–thaw cycles. The CK group showed a sharp decrease while cycles prolonged with less than 30% activity remained. After seven freeze–thaw cycles, the activity in the CK, TP, HP1 and HP2 samples reached 0.11, 0.20, 0.18 and 0.19 μmol/mg/min, respectively. Apparently, the incorporation of hydrolysates into bighead carp fillets significantly delayed the protein denaturation. 

The decrease in Ca^2+^-ATPase activity is closely related to the conformation change and aggregation of the myosin head. Due to a large number of low MW peptides, bone hydrolysates from Protamex showed an effect against myosin denaturation that was equivalent to tea polyphenol. One reason for the cryoprotective effect of bone hydrolysates might derive from the hydrophilicity of amino acid residues in peptides that reduce the formation of ice crystals [1]. Moreover, the delayed oxidation of sulfhydryl groups would also inhibit the decrease in Ca^2+^-ATPase activity in the globular head of myosin [3]. Bone hydrolysates showed potential for being effective cryoprotectants in frozen fish fillets.

### 3.4. Effect of Bone Hydrolysates on Lipid Oxidation of Frozen Fish Fillets

**Figure 6:**
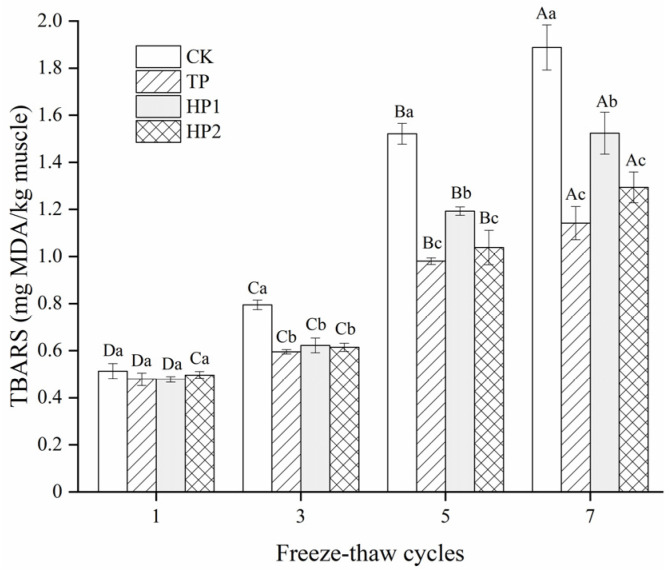
Changes in TBARS of freeze-thawed fish fillets: CK: fillets in distilled water; TP: fillets in tea polyphenol; HP1: fillets in 1% bone hydrolysates; HP2: fillets in 2% bone hydrolysates. Different capital letters indicate that the results of the same treatment differ significantly in freeze–thaw cycles (*p* < 0.05). Different lowercase letters indicate that the results of different treatments differ significantly in the same freeze–thaw cycle (*p* < 0.05).

Multiple freezing/thawing episodes lead to muscle lipid oxidation, which itself facilitates the formation of secondary oxidation products. As shown in Figure 6, the lipid oxidation in fish fillets was present in the form of a TBARS value. The initial TBARS value of fish fillets was below 0.5 mg MDA/kg muscle. With the prolonged freeze–thaw cycles, the TBARS values increased variously in all samples, eventually reaching 1.88 (CK), 1.14 (TP), 1.52 (HP1), and 1.29 (HP2) mg MDA/kg muscle, respectively. Compared with a drastic increase in CK samples (3.76-fold), the TBARS value in the bone hydrolysates-treated fillets reduced 2.58-fold, indicating excellent antioxidant activity against lipid oxidation. Based on their good radical scavenging ability, bone hydrolysates from Protamex with a DH of 10% could effectively postpone lipid oxidation in fish fillets during the freeze–thaw process. 

### 3.5. Physical Quality Changes of Frozen Fish Fillets

#### 3.5.1. Drip Loss

**Figure 7:**
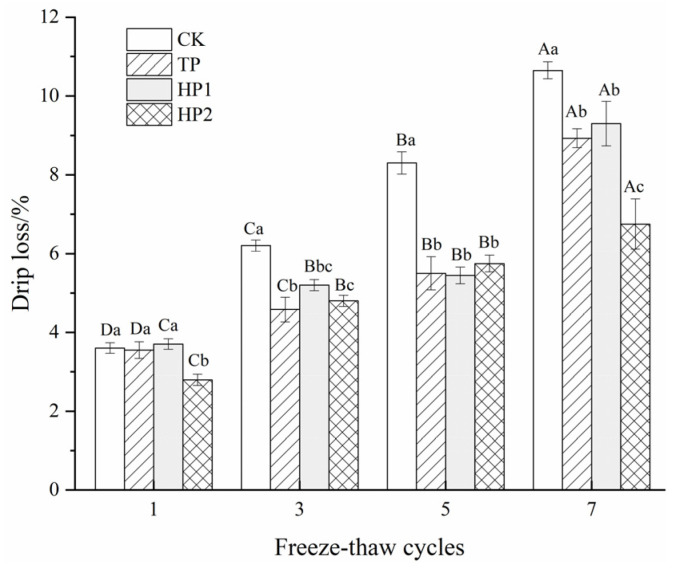
Changes in drip loss of freeze-thawed fish fillets: CK: fillets in distilled water; TP: fillets in tea polyphenol; HP1: fillets in 1% bone hydrolysates; HP2: fillets in 2% bone hydrolysates. Different capital letters indicate that the results differ significantly in freeze–thaw cycles with the same treatment (*p* < 0.05). Different lowercase letters indicate that the results differ significantly with different treatments in the same freeze–thaw cycle (*p* < 0.05).

Drip loss is an indicator of the water-binding capacity of fish fillets. It is associated with the denaturation or aggregation of myofibrillar proteins [13]. The structural changes in myofibrils would negatively affect water holding capacity, leading to an increase in the drip loss of fish fillets. There was no obvious difference (*p* < 0.05) in drip loss in all samples during the first cycle (Figure 7). However, a severe drip loss was found in the control group, with a 7% increase found after multiple freeze–thaw cycles. High drip loss was possibly associated with protein denaturation after multiple freeze–thaw cycles [38]. Drip loss was significantly decelerated by tea polyphenol and bone hydrolysates, with only 5.38%, 5.60% and 3.95% increases for TP, HP1 and HP2, respectively. In addition, the drip loss of fish fillets decreased with increasing doses of bone hydrolysates. This may be attributed to the water-constraining effects of hydrophilic amino acid residues in bone hydrolysates that prevent water migration from forming ice crystals during freeze–thaw cycles [39].

#### 3.5.2. Textural Properties

To evaluate the quality of fillets with different antioxidants, textural parameters (hardness and springiness) were measured. Hardness means the necessary force to deform an object, while springiness means the ability to recover from deformation after an exogenous process [40]. The hardness and springiness values of the fillets decreased gradually in all groups throughout the repeated freeze–thaw cycles (Table 1). The values of hardness and springiness in the CK group decreased to 1.97 N and 0.63 mm after seven freeze–thaw cycles. In contrast, no obvious difference (*p* > 0.05) was observed in hardness value among the treated samples, which finally reached around 2.28 N. Springiness values reached 0.85 and 0.99 mm in the bone hydrolysates-treated group after seven freeze–thaw cycles. In addition, tea polyphenol showed a better protective effect against the decline in springiness, with its value eventually being maintained at 1.21 mm. Compared to the control, bone hydrolysates could effectively delay the decline in texture, which might benefit from the cryoprotective effect of low MW peptides in freeze-thawed fish fillets [31].

In this study, bone hydrolysates effectively inhibited the oxidation of protein and lipids in frozen fish fillets. Compared to some synthetic antioxidants (such as BHT and BHA), fish bone hydrolysates could be an alternative and safe source of natural antioxidants for the improvement of the quality of fish products. Protein hydrolysates with cryoprotective effects might also be used to replace or partially replace traditional cryoprotectants, including the phosphates and sugars found in frozen products [26,41]. However, the full implementation of protein hydrolysates and bioactive peptides as food preservatives or bioactive components is still being by several challenges, such as low potency, unrefined nature and bitter taste [42]. In addition, the safety of protein hydrolysate-based products should be assessed before commercialization.

## 4. Conclusions

The hydrolysis of proteins from bighead carp bone was performed using Protamex and Alcalase with a degree of hydrolysis of 5%, 10% and 15%. Bone hydrolysates of Protamex exhibited stronger free radical scavenging and metal chelating activities in vitro. After being added to fillets through a vacuum impregnation process, bone hydrolysates could effectively retard lipid/protein oxidation and degradation. This was reflected in improvements in texture and drip loss, greater Ca^2+^-ATPase activity, higher sulfhydryl contents, lower carbonyls and TBARS values, even after multiple freeze–thaw cycles. Therefore, bone hydrolysates could serve as alternative antioxidants in frozen products. Further research is needed to elucidate the sequences and structure–activity relationship of antioxidant peptides in fish bone hydrolysates.

## Figures and Tables

**Table 1:** Changes in texture parameters of freeze-thawed fish fillets.

Texture Parameters	Times	CK	TP	HP1	HP2
Hardness (N)	1	2.92 ± 0.47 ^Ab^	3.63 ± 0.07 ^Aa^	3.33 ± 0.18 ^Aa^	3.27 ± 0.06 ^Aa^
	3	2.31 ± 0.09 ^ABc^	3.17 ± 0.13 ^Ba^	3.12 ± 0.15 ^ABa^	2.70 ± 0.13 ^Bb^
	5	2.08 ± 0.14 ^Bb^	2.48 ± 0.12 ^Cab^	2.72 ± 0.21 ^Ba^	2.66 ± 0.18 ^Ba^
	7	1.97 ± 0.06 ^Ba^	2.28 ± 0.05 ^Ca^	2.28 ± 0.09 ^Ba^	2.27 ± 0.11 ^Cb^
Springiness (mm)	1	4.22 ± 0.15 ^Ac^	4.43 ± 0.16 ^Ab^	4.84 ± 0.11 ^Aa^	4.40 ± 0.10 ^Ab^
	3	2.93 ± 0.08 ^Bc^	4.63 ± 0.21 ^Aa^	4.24 ± 0.16 ^Bab^	4.11 ± 0.14 ^Ab^
	5	2.42 ± 0.04 ^Cc^	3.71 ± 0.15 ^Bab^	3.80 ± 0.08 ^Ba^	3.48 ± 0.07 ^Ab^
	7	0.63 ± 0.06 ^Db^	1.21 ± 0.22 ^Ca^	0.85 ± 0.05 ^Ca^	0.99 ± 0.08 ^Ca^

CK: fillets in distilled water; TP: fillets in tea polyphenol; HP1: fillets in 1% bone hydrolysates; HP2: fillets in 2% bone hydrolysates. Different capital letters indicate that the results in freeze–thaw cycles at the same treatment differ significantly (*p* < 0.05). Different lowercase letters indicate that the results in different treatments at the same freeze–thaw cycle differ significantly (*p* < 0.05).

## Data Availability

Data will be available under request.
